# Self-Information-Driven Gated Graph Convolutional Network for Occluded Person Re-Identification

**DOI:** 10.3390/s26092901

**Published:** 2026-05-06

**Authors:** Wanran Guo, Jiake Meng, Yuan Xue, Yaxian Fan, Zhenyu Fang

**Affiliations:** 1 School of Mathematical Sciences, Chengdu University of Technology, Chengdu 610059, China; 13171955930@163.com (W.G.); xueyuan07@cdut.edu.cn (Y.X.); 19980850361@163.com (Y.F.); 17882342474@163.com (Z.F.); 2 Geomathematics Key Laboratory of Sichuan Province, Chengdu 610059, China

**Keywords:** person re-identification, occlusion, graph convolutional network (GCN), self-information, uncertainty modeling, gating mechanism

## Abstract

Occluded person re-identification (Re-ID) aims to accurately match occluded pedestrian images against complete gallery images captured across multiple cameras, a task that is critical to public security and intelligent surveillance systems. Existing graph neural network (GNN)-based methods typically assign uniform aggregation weights to all nodes, failing to reflect the inherent reliability difference between visible and occluded body regions, which allows noise from low-confidence nodes to propagate freely and corrupt the final pedestrian representation. To address this, we propose the Self-Information-Driven Gated Graph Convolutional Network (SI-GCN). Keypoint detection confidence scores are transformed into logarithmic self-information measures as uncertainty priors for a learnable gating mechanism. The proposed SIG module enables visible nodes to dominate information diffusion while occluded nodes absorb more from neighbors, achieving efficient feature updating. A dynamic confidence calibration (DCC) strategy further synchronizes node reliability estimates with feature evolution across successive GCN layers. Extensive experiments on six public benchmarks covering occluded, partial, and holistic Re-ID scenarios demonstrate that SI-GCN achieves state-of-the-art performance, with Rank-1 accuracy and mAP improvements of 1.2% and 0.9%, respectively, over the strongest baseline on the Occluded-REID dataset, demonstrating its strong potential for deployment in real-world public security and urban surveillance applications where occlusion is pervasive.

## 1. Introduction

Person re-identification (Re-ID) aims to retrieve specific individuals across time and multiple cameras and has been widely used in video surveillance, security, and smart cities [1]. However, in real-world deployment, limited camera fields of view, mutual occlusions between pedestrians, and scene obstacles result in a large number of captured images only showing partial regions of the target pedestrian body. How to achieve accurate cross-camera matching under severe feature missing conditions remains an important and unsolved challenge [2]. Therefore, occlusion is widely recognized as the primary bottleneck restricting the reliable deployment of Re-ID systems in real-world scenarios [3].

Over the past decade, the rapid development of deep convolutional neural networks (CNNs) has significantly boosted the overall performance of Re-ID [1]. CNN-based methods extract global discriminative features of images through end-to-end learning and achieve remarkable results on standard benchmarks with the help of metric learning loss functions [4]. To address occlusion and pose variations, part-level methods divide the human body into several regions or stripes to extract features separately and then fuse them with global features to enhance detailed perception. Nevertheless, both types of methods lack an explicit mechanism to distinguish the feature reliability of different regions when handling severe occlusion, resulting in the equal treatment of noisy and effective features, which severely interferes with the final matching decision.

Compared with CNNs, graph neural network (GNN)-based methods possess stronger structure awareness and contextual reasoning abilities by modeling the topological relationships among human keypoints [5]. In the Re-ID task, Zhang et al. [6] proposed the Part-Guided Graph Convolution Network (PGCN) to mine part relationships across images via graph convolution; Lian et al. [7] presented the Local Relation-Aware Graph Convolutional Network (LRGCN) to achieve the adaptive aggregation of local features by constructing overlapping graphs and similarity graphs. These methods effectively improve the model’s robustness to pose variations and partial occlusion, representing the mainstream paradigm of current graph-based Re-ID approaches.

**Figure 1:**
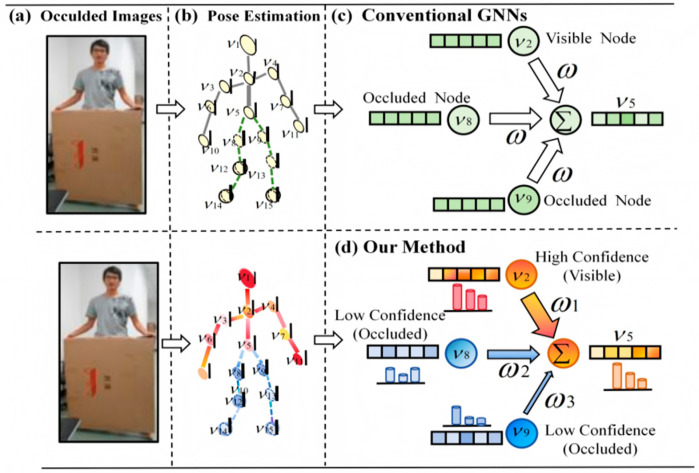
Comparison of information propagation mechanisms between traditional GNN and our proposed SI-GCN. (**a**) Occluded Images: Examples of input images with varying degrees of occlusion, where certain key regions (e.g., joints or object parts) are partially or fully invisible. (**b**) Pose Estimation: Corresponding pose estimation results, depicting the skeletal structure or keypoints of the human body/object derived from the occluded images. (**c**) Uniform propagation: All nodes are treated equally, and noise from low-confidence regions is diffused. (**d**) Self-information-driven gated graph convolution for adaptive propagation: High-confidence nodes (orange) output strongly; low-confidence nodes (blue) suppress output and aggregate contextual information. The thickness of the arrows indicates the information intensity.

However, existing GNN methods suffer from two limitations in their information propagation mechanism. First, they assign equal aggregation weights to all nodes during message passing, failing to reflect the inherent differences in feature reliability between visible nodes and occluded nodes, leading to the diffusion of noise from low-confidence nodes in the graph structure. Second, they lack a reliability-aware weighting mechanism in the feature fusion stage, implicitly treating the contributions of all nodes to the final identity embedding as equal, which further weakens the model’s robustness. As shown in Figure 1a, classical GNNs treat high-confidence nodes (orange; light color denotes low uncertainty) and low-confidence occluded nodes (blue; dark color denotes high uncertainty) equally during information propagation and cannot effectively suppress noise diffusion.

To address the above issues, this paper proposes the Self-Information-Driven Gated Graph Convolutional Network (SI-GCN). As illustrated in Figure 1b, SI-GCN converts the keypoint detection confidence output by the pose estimator into a self-information metric, which drives learnable gating coefficients to perform the asymmetric adaptive regulation of the information flow between nodes. Low-confidence occluded nodes actively aggregate semantic information from high-confidence neighbors to repair features while suppressing the outward propagation of their own noise. High-confidence visible nodes strengthen the dominant roles of their features with larger weights to avoid over-smoothing. In addition, the dynamic confidence calibration (DCC) strategy enables node reliability estimation to be updated synchronously with feature evolution across multi-layer graph convolutions, further enhancing the framework’s continuous perception of changing occlusion states.

The main contributions of this paper are summarized as follows:

(1) We propose the Self-Information-Driven Gated Graph Convolutional Network (SI-GCN) framework. From an information-theoretic perspective, we revisit the node feature propagation mechanism under occlusion, convert keypoint detection confidence into logarithmic self-information as an uncertainty prior, and construct an adaptive graph convolution learning framework for occluded person Re-ID.

(2) We design the self-information gated graph convolution module (SIG) and the dynamic confidence calibration (DCC) strategy. SIG realizes asymmetric information flow regulation between nodes via learnable gating coefficients, effectively suppressing noise diffusion in the graph structure; DCC allows confidence information to propagate and update synchronously with feature information across graph layers, strengthening the framework’s continuous perception of occlusion states.

(3) Extensive experiments on six public benchmark datasets covering occluded, partial, and holistic Re-ID scenarios demonstrate that SI-GCN achieves competitive performance. In particular, on the Occluded-REID dataset, its Rank-1 accuracy and mAP surpass those of the current strongest baseline by 1.2% and 0.9%, respectively.

The remainder of this paper is organized as follows. Section 2 reviews related works, covering CNN-based Re-ID methods, GNN-based Re-ID methods, and information-theoretic modeling approaches for graph neural networks. Section 3 presents the proposed SI-GCN framework in detail, including the semantic topology module (STM), the self-information gated graph convolution module (SIG), the dynamic confidence calibration (DCC) strategy, and the loss function design. Section 4 describes the experimental setup, reports quantitative comparisons with state-of-the-art methods on six public benchmarks, and provides ablation studies and a model analysis. Section 5 concludes the paper and outlines directions for future work.

## 2. Related Works

Regarding CNN-based methods for Re-ID, the rise of deep convolutional neural networks (CNNs) has significantly boosted the performance of person re-identification and achieved remarkable progress on multiple standard benchmarks [1,8]. Early methods mainly relied on global feature learning. He et al. [9] introduced the Transformer architecture into Re-ID and proposed TransReID, which enhances global context modeling via a patch-based strategy and lateral information embedding. Min et al. [10] proposed a dual-stream interactive learning framework based on feature reconstruction, which provides more challenging training samples for metric learning through a correlation graph sampler (CGS). Meanwhile, they designed a global sparse attention network (GSANet) to strengthen the feature representation ability of the backbone, establishing an intrinsic correlation and collaborative optimization mechanism between metric learning and representation learning.

To further improve the model’s perception of local details, researchers introduced part-level feature exploration into CNN-based frameworks [4]. Sun et al. [4] proposed the PCB network, which generates multiple local stripes via the uniform horizontal division of feature maps and constrains the feature learning of each stripe with independent classifiers, significantly improving the fine granularity of representations. Huang et al. [11] attached a lightweight part segmentation head to the backbone network for multi-task training, guiding feature representation to focus on diverse semantic body regions. However, when dealing with severe occlusion, the above methods lack an explicit mechanism to distinguish the feature reliability of different local regions. Noisy features from occluded regions are treated equally with valid features, which restricts the matching accuracy of the model in real occlusion scenarios.

Compared with CNNs, graph neural network (GNN)-based methods possess stronger structure awareness and contextual reasoning abilities by modeling topological relationships among human keypoints, and they have attracted extensive attention in recent years [12]. Zhang et al. [6] proposed the Part-Guided Graph Convolution Network (PGCN), which constructs a local part graph and fully exploits the relationships between different body parts and across images via graph convolution. Zhu et al. [13] focused on cross-resolution scenarios, generated human topological graphs using keypoints, and performed multi-scale neighborhood information aggregation through a two-layer graph convolution network to suppress background interference. Lian et al. [7] proposed LRGCN, which achieves the adaptive aggregation of local features by constructing overlapping graphs and similarity graphs, effectively improving the model’s robustness to pose variations and partial occlusion.

Nevertheless, these methods [6,7,13] lack the ability to dynamically adjust the information flow paths and intensities according to the graph context, thereby limiting the generalization ability of the model to a certain extent.

Regarding information-theoretic modeling methods for GNNs, information-theoretic methods provide an effective theoretical tool for modeling feature uncertainty in graph neural networks, and they have been preliminarily explored in the Re-ID field in recent years. Chen et al. [14] reduced the negative impact of camera variations on unsupervised person re-identification by minimizing the mutual information between pedestrian features and camera features. Liao et al. [15] proposed an entropy model for graph neural networks, which evaluates node importance through an entropy calculation framework that fuses node attributes and the topological structure.

However, most existing methods require an external entropy measurement module, leading to a high computational overhead. Moreover, these methods fail to fully exploit keypoint detection confidence as an endogenous prior for node feature reliability, resulting in the underutilization of node uncertainty information under occlusion.

Therefore, this paper proposes the Self-Information-Driven Gated Graph Convolutional Network (SI-GCN). By explicitly modeling node uncertainty and designing an adaptive gating unit, dynamic filtering and enhancements in information flow are realized. The detailed network architecture of this mechanism will be elaborated upon in Section 3.

## 3. The Proposed Method

### 3.1. Overview

**Figure 2:**
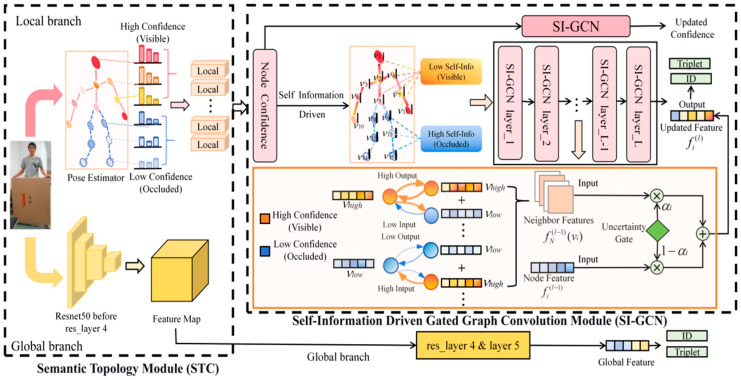
Framework of SI-GCN. For an input occluded pedestrian image, the global branch uses ResNet-50 to extract holistic semantic features. The local graph branch adopts HRNet for pose estimation to construct a human keypoint graph and then performs structure-aware feature extraction via stacked self-information gated graph convolution layers. The dynamic confidence calibration mechanism gradually updates the node confidence during graph propagation. The two branches are jointly optimized end-to-end under the supervision of the identity classification loss and hard triplet loss.

In this paper, we propose the Self-Information-Driven Gated Graph Convolutional Network (SI-GCN) for occluded person re-identification. The framework models node uncertainty from an information-theoretic perspective. It incorporates a learnable gated graph convolution mechanism to suppress noisy feature diffusion and improve reliability awareness. Figure 2 illustrates the complete network architecture and training pipeline. SI-GCN comprises four components: a shared backbone network, a semantic topology module (STM), a self-information gated graph convolution module (SIG), and a dynamic confidence calibration (DCC) strategy. Specifically, the STM constructs a structured human keypoint graph by fusing visual features with body topology priors, providing a reliable graph representation for subsequent feature learning. Building upon this graph structure, SIG performs adaptive information propagation by leveraging self-information-driven gating coefficients to regulate the feature flow between nodes. DCC further refines the node reliability estimates layer by layer as features evolve through successive graph convolution operations. Section 3.2 details the construction of the STM. Section 3.3 introduces the self-information metric and the gated node feature update mechanism of SIG. Section 3.4 describes the dynamic confidence calibration scheme of DCC. Section 3.5 elaborates on the loss functions adopted in this framework.

Given an input occluded pedestrian image, the first four residual stages of the backbone network first perform multi-scale feature extraction. The output intermediate feature maps are then fed into both the global and local graph branches simultaneously for discriminative feature learning. The global branch retains the last residual stage of the backbone network to conduct deep semantic encoding on the intermediate feature maps and generates a compact global appearance descriptor via generalized mean pooling (GeM). The local graph branch adopts the pre-trained HRNet-W32 as a pose estimator to extract semantic keypoint coordinates and their detection confidences, thereby constructing a structured graph representation with human body topology priors. Fine-grained structural feature learning is accomplished through stacked SIG layers, during which the DCC strategy synchronously updates the confidence estimation of each node layer by layer. Finally, the features output by the two branches are concatenated along the channel dimension to form the pedestrian identity descriptor in the testing phase. The entire network is trained end to end under the joint supervision of the identity classification loss and hard triplet loss.

It is worth noting that the global branch of SI-GCN provides an implicit architectural fallback that is entirely decoupled from the keypoint detection confidence. Following the complementary dual-branch paradigm adopted in recent Re-ID works [16], the global branch encodes holistic appearance features directly from the backbone via generalized mean pooling, producing a confidence-agnostic identity descriptor that is unaffected by pose estimator failures. Since the final pedestrian representation is formed by the channel-wise concatenation of both branch outputs, the global branch acts as a stable semantic anchor that bounds the impact of local graph-branch degradation on the overall matching accuracy, regardless of whether this degradation originates from low-confidence occlusion or confidently incorrect keypoint localization.

### 3.2. Semantic Topology Module (STM)

When the target pedestrian is occluded, the accurate perception of the feature reliability of each local region plays a critical role. This motivates us to explicitly fuse visual features with the human body topology to construct a structure-aware local graph representation, rather than relying on heatmap masks for soft weighting. For a given pedestrian image, the local feature branch extracts the intermediate feature map $X\in R^{H\times W\times C}$ from the output of the fourth residual stage of the backbone network, where H,W and C represent the height, width and number of channels of the feature map $X\in R^{H\times W\times C}$, respectively. The pre-trained HRNet-W32 pose estimator processes the input image in parallel with the backbone network and outputs the set of keypoint coordinates $P={\left\{\left(x_{i},y_{i}\right)\right\}}_{i=1}^{K}$ and the set of detection confidences $C={\left\{c_{i}\right\}}_{i=1}^{K}$. Following the human body structure standard [17], this paper defines K = 17 semantic keypoints, covering key joints such as the head, shoulders, elbows, wrists, hips, knees and ankles. The initial node feature at each key point $\left(x_{i},y_{i}\right)$ is obtained through bilinear interpolation sampling:

(1) $$f_{i}^{\left(0\right)}=\sum_{\left(h,w\right)\in N_{grid\left(x_{i},y_{i}\right)}}\beta \left(h,w,x_{i},y_{i}\right)\cdot X_{h,w}$$

where $N_{grid}$ denotes the four grid points around the coordinate, and β represents the interpolation coefficient.

The global branch aims to preserve the strong representation capabilities of the backbone network for holistic appearance information. The intermediate feature map X is fed into the last residual stage for deep semantic extraction. To obtain a compact and robust global descriptor, generalized mean pooling (GeM) is employed instead of global average pooling (GAP) or global max pooling (GMP), as it effectively captures salient local responses while suppressing background noise interference. The global feature vector $f_{global}$ is defined as

(2) $$f_{global}=R_{global}\left(GeM\left(F_{res5}\left(X\right)\right)\right)$$

where $F_{res5}$ denotes the network operations of the fifth stage, and $R_{global}$ is a dimension reduction module composed of convolution, batch normalization (BN) and ReLU, which is used to compress the feature dimension to 2048.

The set of discrete node features $\nu =\left\{f_{1}^{\left(0\right)},\ldots ,f_{K}^{\left(0\right)}\right\}$ obtained by sampling is organized into structured graph data $G=\left(\nu ,\varepsilon \right)$. The adjacency matrix $A\in {\left\{0,1\right\}}^{K\times K}$ is defined according to the natural skeleton topology of the human body, explicitly modeling two types of edge connections: (1) physical connections $E_{skeleton}$, which link physiologically adjacent joint pairs (e.g., shoulder joint–elbow joint); (2) symmetric connections $E_{sym}$, which connect left–right symmetric parts of the human body (e.g., left shoulder–right shoulder) to facilitate feature interaction across the body axis. In addition, self-loop connections are added to the adjacency matrix to preserve the nodes’ own features. The adjacency matrix is formulated as

(3) $$A_{ij}=\left\{\begin{array}{l} 1,\;if\left(i,j\right)\in E_{skeleton}\cup E_{sym}\;or\;i=j\\ 0,\;otherwise \end{array}\right.$$

### 3.3. Self-Information-Driven Gated Graph Convolution (SIG) Module

Classic GNNs adopt uniform aggregation weights for nodes with different reliabilities, which inevitably leads to the diffusion of noisy features across the graph structure. To address this issue, we revisit the node feature propagation mechanism from the perspective of information theory and propose the self-information-driven gated graph convolution (SIG) module. By introducing a self-information measure based on the detection confidence, we implement the differentiated and adaptive regulation of the information flow between nodes.

#### 3.3.1. Node Self-Information Construction

The pose estimator outputs a detection confidence $c_{i}\in \left(0,1\right)$ for each keypoint node $v_{i}$ in the graph, which directly reflects the visibility of each part of the human body in the image. However, directly using the confidence as a linear weight to scale the node features has two shortcomings: (1) the linear mapping has a weak gradient response in the high-confidence interval and is insensitive to subtle reliability differences between visible nodes; (2) its numerical dimension is heterogeneous with node features, making it difficult to drive the subsequent learnable gating mechanism within a unified framework. Therefore, we convert the confidence $c_{i}$ into a self-information measure $E_{i}$ through logarithmic transformation, which serves as the uncertainty prior input for the gating mechanism:

(4) $$E_{i}=-\ln\left(c_{i}+\epsilon \right)$$

where ϵ is a small constant to prevent numerical overflow (set to $e^{-6}$ in this paper).

#### 3.3.2. Node Feature Update

**Figure 3:**
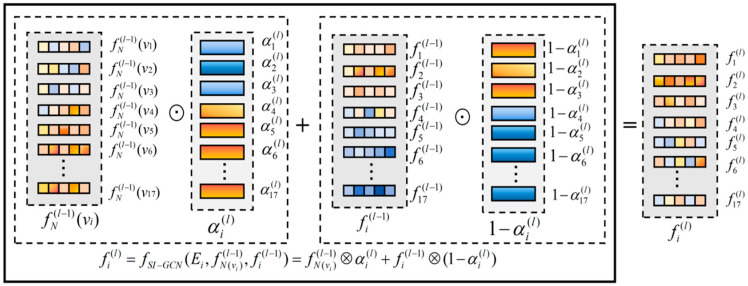
Detailed schematic of the self-information-driven gating mechanism (SIG) within the GCN layer. The node detection confidence is converted into self-information via logarithmic transformation, which, together with node features, drives the learnable gating coefficient $\alpha_{i}^{\left(l\right)}$ to perform the weighted fusion of the neighborhood aggregated features and the self-features. Orange blocks correspond to high-confidence visible nodes (small $\alpha_{i}^{\left(l\right)}$), which dominate their own features. Blue blocks correspond to low-confidence occluded nodes (large $\alpha_{i}^{\left(l\right)}$), which actively aggregate neighborhood semantics to repair local features.

Based on the above self-information metric, this section designs an asymmetric adaptive information propagation mechanism, as illustrated in Figure 3. For a given graph G, let the feature of node $v_{i}$ at the l−1-th layer be $f_{i}^{\left(l-1\right)}$; the SIG module performs the following gating updates in parallel for all semantic nodes in the graph:

(5) $$f_{i}^{\left(l\right)}=f_{SI-GCN}\left(E_{i},f_{N\left(v_{i}\right)}^{\left(l-1\right)},f_{i}^{\left(l-1\right)}\right)=\alpha_{i}^{\left(l\right)}⊙f_{N\left(v_{i}\right)}^{\left(l-1\right)}+\left(1-\alpha_{i}^{\left(l\right)}\right)⊙f_{i}^{\left(l-1\right)}$$

where $f_{SI-GCN}\left(\cdot \right)$ denotes the update function driven by self-information, and $\alpha_{i}^{\left(l\right)}$ represents the uncertainty gating coefficient of the current node $v_{i}$ at the l-th layer. The above equation unifies the retention of the node’s own features and the absorption of neighborhood information in the form of continuous interpolation: the gating coefficient increases with an increase in node uncertainty. For high self-information nodes (occluded nodes), larger neighborhood aggregation weights are assigned to achieve feature restoration while suppressing the outward propagation of their noise. For low self-information nodes (visible nodes), the dominant role of their own features is strengthened to prevent the loss of discriminative information caused by over-smoothing.

#### 3.3.3. Gating Coefficient Calculation

The uncertainty gating coefficient $\alpha_{i}^{\left(l\right)}$ is jointly driven by the node features at the current layer and the self-information. They are concatenated and fed into the gating network, enabling it to simultaneously perceive the semantic content of the current features and the confidence-driven uncertainty level:

(6) $$\alpha_{i}^{\left(l\right)}=\sigma (W_{gate}\left[\left.f_{i}^{\left(l-1\right)}\|E_{i}\right]+b_{gate}\right)$$

where $\alpha_{i}^{\left(l\right)}\in \left(0,1\right)$ controls the intensity of neighborhood information flowing into the current node, $\sigma \left(\cdot \right)$ denotes the Sigmoid activation function, $W_{gate}$ represents the learnable weight matrix, $b_{gate}$ denotes the learnable bias term, and [⋅‖⋅] indicates the channel concatenation operation.

#### 3.3.4. Neighborhood Aggregation Weights

The neighborhood aggregated feature $f_{N\left(v_{i}\right)}^{\left(l-1\right)}$ is obtained via the reliability-aware weighted summation of the neighbor node features:

(7) $$\left\{\begin{array}{l} f_{N\left(v_{i}\right)}^{\left(l-1\right)}=\sum_{j\in N\left(i\right)}\gamma_{ij}\cdot W_{agg}f_{j}^{\left(l-1\right)}\\ \gamma_{ij}=soft\max\left(1-\alpha_{j}^{\left(l-1\right)}\right)=\frac{\exp\left(1-\alpha_{j}^{\left(l-1\right)}\right)}{\sum_{k\in N\left(i\right)}\exp\left(1-\alpha_{k}^{\left(l-1\right)}\right)} \end{array}\right.$$

where $N\left(i\right)$ denotes the neighbor set of node $v_{i}$, and $W_{agg}$ is the learnable feature transformation matrix. Equation (7) is used to calculate the normalized aggregation weights based on the gating coefficients of neighboring nodes.

It is important to acknowledge the inherent limits of the feature completion mechanism realized by the SIG module. The aggregation process assumes that at least a subset of neighboring nodes retains sufficient reliability to serve as a semantic reference for occluded nodes, an assumption that holds in typical partial occlusion scenarios but breaks down when large contiguous body regions are simultaneously missing. In such cases, multiple adjacent low-confidence nodes become mutually dependent for feature completion, producing a circular aggregation regime analogous to feature hallucination in generative models. Two design choices partially mitigate this risk. First, unlike linear confidence scaling, the logarithmic self-information transformation non-linearly amplifies uncertainty for severely occluded nodes, asymmetrically suppressing their outward feature propagation within co-occluded clusters. Second, the global branch provides a stable, graph-independent identity representation that anchors the final descriptor when the local graph branch enters an underdetermined completion regime. Nevertheless, we acknowledge that these mitigations do not eliminate the fundamental limitation under extreme occlusion, and we consider this in detail in the discussion of the limitations in Section 6.

### 3.4. Dynamic Confidence Calibration Strategy

During multi-layer message passing, we observe that (1) after continuously absorbing semantic information from high-confidence neighboring nodes, the feature representations of occluded nodes are gradually repaired layer by layer, and their actual reliability is improved compared with the initial pose estimation confidence; (2) if only the initial confidence output by the pose estimator is used as a static prior, it cannot accurately reflect the actual reliability state of deep features, which further leads to a systematic deviation between the gating decision of each layer and the real uncertainty of nodes. Based on the above observations, this paper designs a dynamic confidence calibration (DCC) strategy, which enables the confidence estimation of each node to be synchronously propagated and calibrated with feature information across graph layers, ensuring that the gating decision of each layer is based on the latest node reliability state of the current layer. The dynamic update process of the confidence of node $v_{i}$ from the l−1-th layer to the l-th layer is defined as follows:

(8) $$\left\{\begin{array}{l} c_{i}^{\left(l\right)}=f_{SI-GCN}\left(E_{i},c_{N\left(v_{i}\right)}^{\left(l-1\right)},c_{i}^{\left(l-1\right)}\right)=\alpha_{i}^{\left(l\right)}\cdot c_{N\left(v_{i}\right)}^{\left(l-1\right)}+\left(1-\alpha_{i}^{\left(l\right)}\right)\cdot c_{i}^{\left(l-1\right)}\\ c_{N\left(v_{i}\right)}^{\left(l-1\right)}=\frac{1}{\left|N\left(i\right)\right|}\sum_{j\in N\left(i\right)}c_{j}^{\left(l-1\right)} \end{array}\right.$$

where $c_{i}^{\left(l-1\right)}$ is the confidence of the node itself from the previous layer, $c_{N\left(v_{i}\right)}^{\left(l-1\right)}$ is the average confidence of its neighboring nodes, and $\alpha_{i}^{\left(l\right)}$ is the uncertainty gating coefficient defined earlier.

### 3.5. Loss Function

To optimize the proposed framework, a joint supervision scheme consisting of the identity classification loss and hard triplet loss is adopted during the training phase, which is applied to the global branch and the local graph branch, respectively. The overall loss function is defined as follows:

(9) $$L_{total}=\left(L_{id}^{global}+L_{tri}^{global}\right)+\lambda \left(L_{id}^{graph}+L_{tri}^{graph}\right)$$

where the weight parameter λ is used to balance the loss contributions of the two branches, and the specific sensitivity analysis is detailed in Section 4.

Among them, the expression of the identity classification loss $L_{id}$ is

(10) $$L_{id}=-\sum_{i=1}^{P\times K}\log\frac{e^{W_{y_{i}}^{T}x_{i}}}{\sum_{c=1}^{N_{id}}e^{W_{c}^{T}x_{i}}}$$

In this loss function, $N_{id}$ denotes the total number of identities in the entire training set; W represents the weight parameters of the fully connected (FC) classification layer; $y_{i}$ and $x_{i}$ are the ground-truth identity label and the input feature of the classification layer for the i-th image, respectively. In this paper, the ID loss is calculated separately for the features of the global branch and the local graph branch to ensure that both branches can extract semantically discriminative features.

The hard triplet loss $L_{tri}$ differs from the standard triplet loss. It aims to maximize the distance between the hardest negative sample pairs within a batch, while constraining the distance between the hardest positive sample pairs with a predefined threshold, thus forcing the network to learn more discriminative feature embeddings. In a mini-batch containing P×K images (i.e., randomly sampling P identities with K images per identity), the hard triplet loss is defined as follows:

(11) $$L_{tri}=\sum_{i=1}^{P}\sum_{a=1}^{K}{\left[m+\underset{p=1\ldots K}{\max}\|f_{i,a}-f_{i,p}{\|}_{2}-\underset{\begin{array}{c} j=1\ldots P\\ n=1\ldots K\\ j\neq i \end{array}}{\min}\|f_{i,a}-f_{i,p}{\|}_{2}\right]}_{+}$$

where $f_{i,a}$, $f_{i,p}$ and $f_{j,n}$ denote the feature vectors of the anchor sample, positive sample and negative sample, respectively; m=0.3 is the predefined distance margin. The hard triplet loss is applied to the outputs of both branches simultaneously to further enlarge the inter-class distance of local topological features.

With the joint supervision of the identity classification loss and the hard triplet loss applied to both branches, the entire SI-GCN framework is trained end to end. In the following section, we describe extensive experiments on multiple public benchmarks to evaluate the effectiveness of the proposed framework.

## 4. Experiments

### 4.1. Datasets and Evaluation Measures

To verify the effectiveness of our method for occluded Re-ID, experiments are conducted on six public benchmark datasets, covering three evaluation scenarios: occluded Re-ID (Occluded-Duke [18], Occluded-REID [19]), partial Re-ID (Partial-REID [20], Partial-iLIDS [21]) and holistic Re-ID (Market-1501 [22], DukeMTMC-reID [23]). For all datasets, the query set refers to the collection of probe images used to search for matching identities across cameras; the gallery set refers to the candidate image pool against which each query is matched; and images per identity denotes the number of images available for each individual pedestrian across all cameras.

Occluded-Duke [18] is selected from the DukeMTMC-reID dataset, retaining images with occlusions and filtering out partially overlapping ones. It consists of 15,618 training images, 17,661 gallery images and 2210 occluded query images.

Occluded-REID [19] was collected using mobile device cameras and contains 2000 images of 200 occluded pedestrians. Each identity has five full-body images and five severely occluded images with different occlusion types.

Partial-REID [20] is a specially designed partial person dataset including 600 images of 60 individuals, with five full-body images and five partial images per person. The images were collected on a university campus under varying viewpoints, backgrounds and severe occlusion types. All probe images are occluded, while all gallery images are holistic.

Partial-iLIDS [21] is a simulated partial re-identification dataset based on the iLIDS dataset. The iLIDS dataset contains 476 images of 119 people captured by multiple non-overlapping cameras.

Market-1501 [22] contains a few occluded pedestrian images and can serve as a holistic Re-ID dataset. It comprises 32,668 images of 1501 subjects captured by six cameras.

DukeMTMC-reID [23] contains 1404 identities, 16,522 training images, 2228 queries and 17,661 gallery images. Although occluded images exist, holistic images are far more numerous, so DukeMTMC-reID has been treated as a holistic Re-ID dataset in previous works.

Regarding the evaluation metrics, following standard evaluation protocols in person re-identification, we adopt two complementary metrics: Rank-1 accuracy and the mean average precision (mAP). Rank-1 accuracy is derived from the cumulative matching characteristic (CMC) curve, which reports the probability that a correct match appears within the top-k positions of the ranked gallery list. At the Rank-1 level, this reduces to evaluating whether the top-retrieved result is a true match, providing the most stringent measure of the retrieval precision. Mean average precision (mAP) reflects the area under the precision–recall curve averaged over all query identities, capturing both the precision and completeness of retrieval across the full ranking—a metric that is particularly sensitive to the ability of the system to retrieve all correct gallery matches rather than only the highest-ranked one. Together, Rank-1 and mAP provide complementary perspectives on system performance: Rank-1 reflects the single-shot retrieval accuracy, while mAP reflects the holistic retrieval quality. All experiments are conducted under the single-query setting.

### 4.2. Implementation Details

**Model Architecture:** For the CNN backbone, we employ the ImageNet-pre-trained ResNet-50, remove its final global average pooling layer and fully connected layer and modify the downsampling stride of the last residual stage from 2 to 1 to preserve spatial feature maps with a higher resolution. The classifier adopts a bottleneck structure consisting of a batch normalization layer, a linear layer and the Softmax function. For the human keypoint model, we use the COCO-pre-trained HRNet-W32 to extract K = 17 semantic keypoints and their detection confidences, including the head, shoulders, elbows, wrists, hips, knees and ankles. The semantic graph is directly constructed based on all original keypoints.

**Training Details:** All input images are resized to 256 × 128 pixels. Standard data augmentation strategies are adopted, including random horizontal flipping, random cropping with 10-pixel padding and random erasing. Color jitter is additionally introduced for occluded datasets to alleviate domain bias. The network is jointly trained end to end for 120 epochs with a batch size of 64, using the PK sampling strategy, which randomly samples P = 16 identities with K = 4 images per identity per mini-batch, where images per identity denotes the number of training images sampled for each pedestrian identity within a batch. The Adam optimizer is utilized, with the learning rate linearly warmed up in the first 10 epochs and decayed to one-tenth of the original value at the 40th and 70th epochs, respectively. The loss balance weight λ is set to 1.0 by default. All experiments are implemented on a single NVIDIA RTX 3090 GPU using the PyTorch 2.0.1 framework.

### 4.3. Experimental Results

#### 4.3.1. Experimental Results on Occluded Re-ID Datasets

Experiments are conducted on Occluded-Duke [18] and Occluded-REID [19] to compare our method with five representative categories, namely holistic Re-ID methods, keypoint-based Re-ID methods, partial Re-ID methods, occluded Re-ID methods and recent Transformer-based Re-ID methods. The results are summarized in Table 1. As can be observed, there is no significant gap between ordinary holistic Re-ID methods and holistic methods with keypoint cues. For instance, PCB [4] and PVPM [24] both achieve around 40% Rank-1 accuracy on Occluded-Duke, indicating that simply employing keypoint information may not substantially benefit the occluded Re-ID task. For partial Re-ID and occluded Re-ID methods, both achieve remarkable improvements on occluded datasets. For example, PAT [25] and PGGA [26] obtain 81.6% and 79.5% Rank-1 accuracy on Occluded-REID, respectively. This demonstrates that partial and occluded tasks share similar challenges, namely learning discriminative features and feature alignment. Transformer-based methods generally demonstrate strong performance on occluded benchmarks, as evidenced by HEFormer [27] and FRCE [28], which achieve 73.2% and 72.4% Rank-1 on Occluded-Duke, respectively, by leveraging large-scale pre-trained ViT backbones. Our approach also has such characteristics: built upon a standard ResNet-50 backbone without ViT pre-training, SI-GCN achieves competitive performance with a significantly lower computational overhead. More importantly, on Occluded-REID, SI-GCN surpasses all Transformer-based competitors, with 86.9% Rank-1 and 79.8% mAP, outperforming FRCE [28] by 2.5% in Rank-1. This demonstrates that explicit uncertainty-aware graph reasoning, which assigns differentiated reliability to visible and occluded nodes rather than treating them uniformly, provides complementary occlusion robustness beyond what global attention mechanisms can achieve under diverse and severe occlusion conditions, validating the effectiveness of the proposed SI-GCN framework.

#### 4.3.2. Experimental Results on Partial Re-ID Datasets

Partial images frequently occur due to imperfect occlusion detection, outliers in camera views and other factors. To further evaluate the proposed framework, we compare it with several state-of-the-art partial-based methods on two partial datasets, Partial-REID [20] and Partial-iLIDS [21], including general partial methods and keypoint-based partial methods. The comparison results are summarized in Table 2. Since the two partial datasets are too small, we use Market-1501 [22] as the training set and the two partial datasets, namely Partial-REID and Partial-iLIDS, as the test sets. As can be observed from Table 2, the proposed framework significantly outperforms other methods by at least 1.6% and 1.4% in terms of the Rank-1 score on Partial-REID and Partial-iLIDS, respectively.

#### 4.3.3. Experimental Results on Holistic Re-ID Datasets

Although recent occluded/partial Re-ID methods have achieved remarkable progress in specific scenarios, they often fail to obtain satisfactory performance on holistic datasets. This is caused by overfitting or noise interference introduced by additional alignment modules when processing unoccluded images. In this section, we show that our proposed framework can also achieve promising performance on holistic Re-ID datasets, including Market-1501 [22] and DukeMTMC-reID [23]. We compare SI-GCN with three mainstream categories of methods, namely ordinary holistic Re-ID methods, semantic parsing-based Re-ID methods and keypoint-aware holistic Re-ID methods. The experimental results are shown in Table 3. As depicted in Table 3, three ordinary holistic Re-ID methods achieve highly competitive performance. For example, BOT [8] obtains Rank-1 scores of 94.1% and 86.4% on the two datasets. However, holistic Re-ID methods using external cues such as semantic parsing and keypoint information perform worse. This is because most images in holistic Re-ID datasets are of high quality and well detected, and ordinary holistic methods are powerful enough to learn discriminative features. Finally, we propose the SIG module and the dynamic confidence calibration (DCC) strategy, which can suppress noisy feature propagation. With the proposed SIG and DCC layers, our framework also achieves comparable performance on both holistic Re-ID datasets. Specifically, we achieve Rank-1 scores of 94.6% and 88.5% on the Market-1501 and DukeMTMC-reID datasets, respectively.

It is worth noting that SI-GCN does not exhibit a specialization bias toward occluded scenarios, as evidenced by its competitive holistic Re-ID performance on Market-1501 (94.6% Rank-1) and DukeMTMC-reID (88.5% Rank-1) reported in Table 3. Furthermore, the partial Re-ID experiments (whose results are listed in Table 2), where the model is trained on Market-1501 and evaluated on Partial-REID and Partial-iLIDS without fine-tuning, constitute a cross-dataset evaluation, confirming that SI-GCN’s performance gains are generalized across dataset boundaries.

### 4.4. Model Analysis

#### 4.4.1. Analysis of Core Components

This section evaluates the effectiveness of each proposed component in SI-GCN, namely the semantic topology module (STM), the self-information gated graph convolution module (SIG) and the dynamic confidence calibration strategy (DCC). We conduct ablation experiments on the Occluded-Duke and Occluded-REID datasets and report both the Rank-1 accuracy and mAP in Table 4.

Effectiveness of STM. As shown in Index-2, introducing the STM improves the Rank-1 by 5.9% to 48.5% and mAP by 4.5% to 38.2% compared with the baseline model. Here, this baseline (Index-1) is defined as a ResNet-50 global feature branch without any graph-based components, supervised by the identity classification loss and hard triplet loss. Since the STM explicitly grounds node features in semantically meaningful body regions via human body topology priors, it provides a structured representation that enables body-part-aware graph reasoning, which is essential for handling feature misalignment under occlusion.

Effectiveness of SIG. As shown in Index-3, further incorporating SIG yields a substantial improvement of 18.7% in Rank-1 to 67.2% and 18.3% in mAP to 56.5%. Since SIG assigns differentiated gating coefficients derived from self-information measures, it explicitly suppresses noise propagation from low-confidence occluded nodes while enabling targeted semantic aggregation from reliable high-confidence neighbors. Compared with conventional, uniformly weighted GNN aggregation, this asymmetric information flow control provides more discriminative and occlusion-robust node representations, which accounts for the significant performance gain observed in Index-3.

Effectiveness of DCC. As shown in Index-4, adding DCC further improves the performance to 69.8% Rank-1 and 59.2% mAP. Since occluded node features are progressively repaired through deeper GCN layers, their actual reliability improves beyond the static initial pose estimator estimate. DCC dynamically calibrates the node confidence across successive layers, ensuring that gating decisions remain aligned with the evolving feature reliability state and enhancing the framework’s sustained awareness of changing occlusion conditions.

#### 4.4.2. Analysis of Uncertainty Measurement

**Figure 4:**
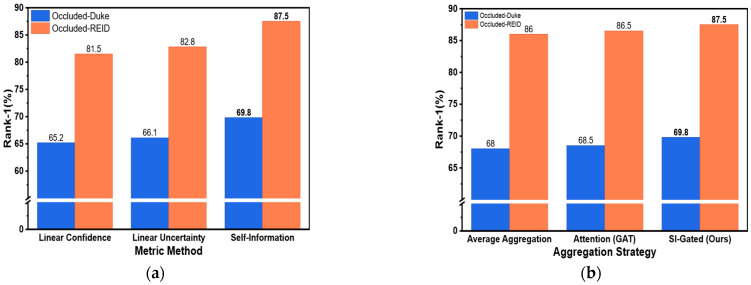
Ablation analysis of key components. (**a**) Performance comparison of different uncertainty metrics in terms of Rank-1 accuracy. (**b**) Performance comparison of neighborhood aggregation strategies in terms of Rank-1 accuracy. Both (**a**,**b**) compare the methods on the Occluded-Duke and Occluded-REID datasets.

To verify the rationality of adopting self-information as the uncertainty quantification metric for node features in the complete SI-GCN framework, this section compares the performance of three metrics on Occluded-Duke and Occluded-REID: the original confidence (linear confidence), linear uncertainty (linear uncertainty) and logarithmic self-information (self-information). As shown in Figure 4a, using self-information achieves the best performance on both datasets. On the Occluded-Duke dataset, it obtains Rank-1 accuracy of 69.8%, outperforming the original confidence and linear uncertainty by 4.6% and 3.7%, respectively. The experimental results demonstrate the effectiveness of our proposed self-information-driven uncertainty metric, which can provide a more discriminative uncertainty prior for the gating mechanism.

#### 4.4.3. Analysis of Neighborhood Aggregation Strategy

A neighborhood aggregation scheme determines the effectiveness of feature completion. We compare three aggregation strategies on the Occluded-Duke and Occluded-REID datasets, namely average aggregation, attention aggregation (GAT) and self-information gated fusion (our SI-GCN). As shown in Figure 4b, although GAT slightly outperforms average aggregation, it still falls significantly behind our method. This indicates that mainstream attention mechanisms rely on feature similarity and tend to mistakenly regard noisy nodes with similar background textures as highly reliable neighbors, thus introducing erroneous contextual information. In contrast, SI-GCN takes node reliability rather than similarity as the prior, accurately suppresses high-uncertainty neighbors through non-linear gating and achieves more robust feature reconstruction.

#### 4.4.4. Parameter Analysis

**Figure 5:**
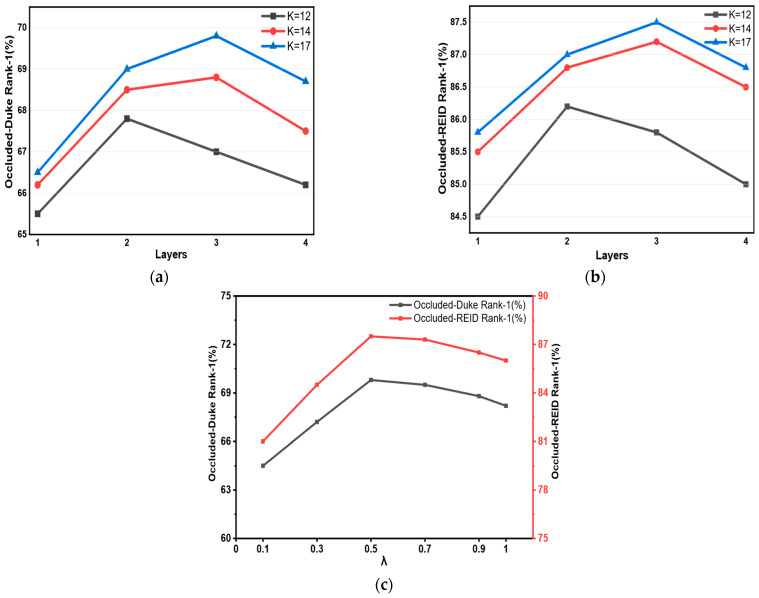
Parameter sensitivity analysis. (**a**,**b**) show the joint effects of the number of keypoints K and the number of GCN layers on the Rank-1 accuracy on the Occluded-Duke (**a**) and Occluded-REID (**b**) datasets. (**c**) presents the analysis of parameter λ in Equation (9). The optimal value is λ = 0.8. The experimental results demonstrate that our model is robust to parameter variations.

In this section, we conduct sensitivity analyses on the number of keypoints K, the number of graph convolution layers L and the loss balancing weight λ. The full-semantic strategy (K = 17) outperforms the head fusion strategy (K = 14) and the torso-only strategy (K = 12) under all layer settings, verifying the effectiveness of preserving the fine-grained facial topology. The optimal network depth increases with the richness of nodes, achieving the best performance at K = 17 and L = 3. When λ varies within the range of $\lambda \in \left\{0.2,0.5,0.8,1.0,1.2,1.5\right\}$, the Rank-1 accuracy reaches its optimum at λ=0.8 on both datasets. Notably, for different values of λ, our model still stably outperforms the baseline models. This indicates that the proposed framework is robust to different weight settings. Note that the performance here, as illustrated in Figure 5, differs from that described in Table 1: in the former case, we achieve 69.8% based on a single run, while, in the latter, this value reaches 68.2%, computed as the average over 10 runs for a fair comparison.

#### 4.4.5. Robustness Analysis Under Confidence Perturbation

**Figure 6:**
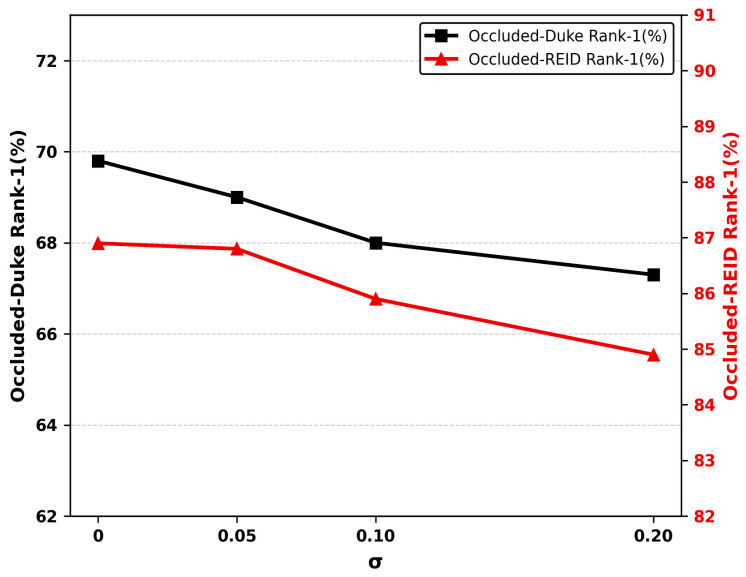
Robustness of SI-GCN to confidence score perturbation. Rank-1 accuracy on Occluded-Duke (left axis, black squares) and Occluded-REID (right axis, red triangles) under zero-mean Gaussian noise of varying standard deviations injected into HRNet confidence scores.

To evaluate the robustness of SI-GCN to inaccurate keypoint confidence scores, we conduct a stress test by injecting zero-mean Gaussian noise of varying standard deviations into all HRNet confidence scores prior to self-information computation. As illustrated in Figure 6, SI-GCN exhibits minor degradation across all perturbation levels on both Occluded-Duke and Occluded-REID. The two datasets display distinct degradation trajectories: Occluded-Duke exhibits a front-loaded pattern with an initial decline of 0.8% at =0.05, while Occluded-REID remains nearly stable at small perturbations (−0.1% at =0.05) before accelerating at larger noise levels. In both cases, the overall performance drop remains within 3% at =0.20, confirming that the self-information gating mechanism retains meaningful discriminability under moderately corrupted uncertainty priors.

### 4.5. Visualization

**Figure 7:**
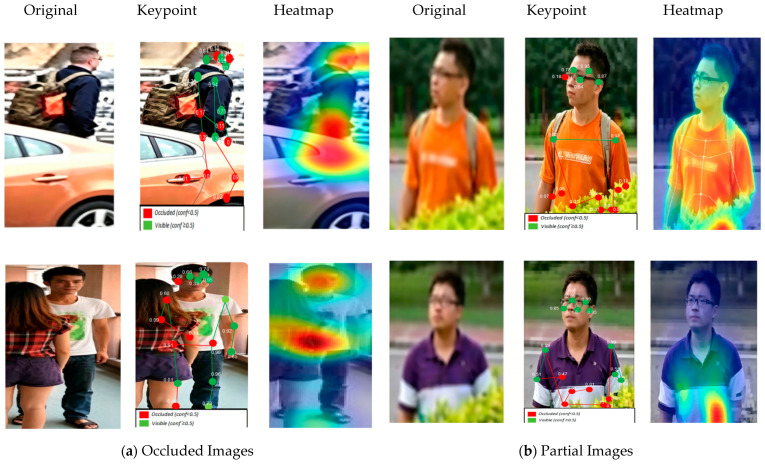
Visualization of gating responses in SI-GCN. From left to right: the original occluded image, the keypoint detection results of HRNet and the self-information gated response heatmap. Warm colors (red) indicate high gating coefficients, corresponding to occluded nodes that actively aggregate neighborhood information; cool colors (blue) indicate low gating coefficients, corresponding to visible nodes that retain their own features.

To intuitively understand SI-GCN, we present a comparative visualization in Figure 7, including the original occluded images, the raw keypoints predicted by HRNet and the gated response heatmaps weighted by self-information. Taking the first occluded image as an example, keypoints on the lower body occluded by obstacles obtain high gating coefficients (highlighted in red), indicating that SI-GCN effectively suppresses the self-noise of occluded nodes during feature aggregation and relies more on neighborhood information for feature repair. This visualization result demonstrates the effectiveness of the adaptive gated graph convolutional network in occluded person re-identification.

### 4.6. Qualitative Results

**Figure 8:**
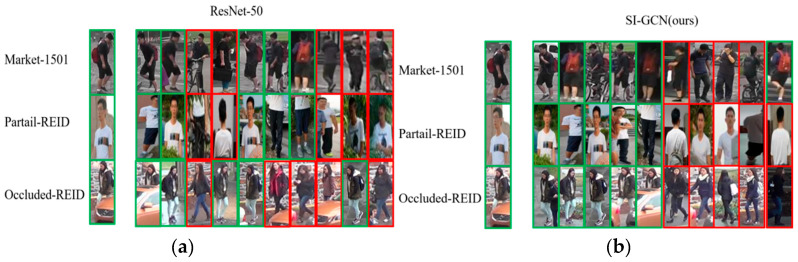
Qualitative comparison of top-10 ranking results between (**a**) the baseline and (**b**) SI-GCN on the Market-1501, Partial-REID and Occluded-REID datasets. Green boxes indicate correct matches, and red boxes indicate incorrect matches.

Figure 8 illustrates the top-10 ranking results of the proposed SI-GCN model and the baseline ResNet-50 on three datasets: Market-1501, Partial-REID and Occluded-REID. The experimental results demonstrate that SI-GCN consistently outperforms ResNet-50 on all these datasets. For instance, on the Partial-REID dataset, the top-5 retrievals of SI-GCN are all correct, whereas ResNet-50 only achieves accuracy of 40%. On Market-1501 and Occluded-REID, the top-5 accuracy of SI-GCN reaches 100%, while HOReID only attains 60% and 80%, respectively. These results highlight the effectiveness and robustness of our SI-GCN framework across different datasets and domain tasks.

## 5. Discussion

The experimental results demonstrate that SI-GCN consistently outperforms existing GNN-based methods across occluded, partial and holistic Re-ID benchmarks, underscoring its effectiveness in addressing the occlusion challenge that is prevalent in real-world pedestrian re-identification systems. As a critical bottleneck in intelligent surveillance and public security applications, occlusion-induced feature corruption remains a key limitation of state-of-the-art Re-ID methods, particularly those relying on graph neural networks (GNNs) for local feature aggregation.

Classical GNN-based methods such as PGCN [6] and LRGCN [7] treat all nodes with uniform aggregation weights, a design that makes them prone to noise diffusion from occluded regions. In contrast, SI-GCN alleviates this issue through a principled information-theoretic mechanism: it quantifies the uncertainty of each node by performing logarithmic self-information transformation on the keypoint detection confidence. This design choice is motivated by the fact that keypoint confidence scores directly encode the reliability of local body regions. Transforming these scores into self-information yields a mathematically rigorous and physically reasonable uncertainty prior, which circumvents the inherent limitations of conventional similarity-based attention mechanisms.

Notably, this uncertainty modeling approach offers distinct advantages over attention-based methods such as PAT [29] and FED [32], which rely solely on feature similarity to weight node contributions. While similarity-based attention mechanisms can effectively highlight discriminative features in unoccluded scenarios, they may mistakenly treat noisy, occluded regions as reliable neighbors, especially in heavy occlusion settings, where feature similarity may be misleading due to partial feature overlap. SI-GCN’s self-information-driven uncertainty prior, by contrast, explicitly encodes the confidence of each node, enabling more robust feature aggregation that prioritizes reliable visible regions while suppressing noise from occluded areas.

Furthermore, the dynamic confidence calibration (DCC) strategy embedded in SI-GCN addresses the limitation of static confidence scores in prior graph-based methods by propagating and updating the node reliability estimates layer by layer. Unlike existing GNN-based Re-ID methods that fix the node confidence at the input stage, the DCC strategy ensures that gating decisions reflect the current feature state at each graph convolution layer, adapting dynamically to the evolving feature representations as they propagate through the network. This dynamic calibration mechanism enhances the model’s adaptability to complex occlusion patterns, where the reliability of local regions may change as features are refined.

Another notable finding is the competitive performance of SI-GCN on holistic datasets, which indicates that the proposed modules do not introduce harmful noise when processing unoccluded images—a common failure mode of alignment-based occluded Re-ID methods. This highlights the model’s strong generalizability, as it can perform consistently well across occluded, partial and holistic Re-ID scenarios.

To contextualize our contributions within the broader Re-ID literature, existing occlusion-aware methods typically fall into the feature alignment and attention-based aggregation categories. Our work differs by introducing an information-theoretic uncertainty modeling framework that unifies keypoint confidence with graph convolution, offering a more principled approach to mitigating noise diffusion than these existing paradigms. The superior performance of SI-GCN across six benchmarks validates this framework’s effectiveness and its potential to advance the state of the art in occluded Re-ID. Overall, SI-GCN provides a viable solution to alleviate occlusion-induced noise diffusion and offers a new perspective for occlusion-aware feature learning with implications for related computer vision tasks, and its strong generalization across diverse scenarios underscores its practical value for real-world surveillance applications.

## 6. Conclusions and Future Work

In this paper, we propose the SI-GCN method to address occluded person re-identification. Specifically, we convert human keypoint confidence into a self-information measure as an uncertainty prior and construct a self-information gated graph convolution module to achieve asymmetric adaptive information propagation between nodes. In addition, we present a dynamic confidence calibration strategy to update node reliability estimation synchronously with graph convolution. Finally, we obtain occlusion-robust and highly discriminative pedestrian feature representations. We comprehensively validate the SI-GCN method on six public datasets covering occluded, partial and holistic scenarios, and the experimental results show that the proposed method outperforms state-of-the-art approaches.

Despite the strong performance demonstrated across six public benchmarks, the proposed SI-GCN framework still has inherent limitations. It relies on an external pose estimator and adopts a fixed anatomical graph topology, which restricts its adaptability to complex occlusion patterns and may lead to unstable inference. In addition, the generalization ability of the self-information gating mechanism to cross-modal and other related tasks has not been systematically verified. To address these issues, we plan future work in three aspects. First, we will explore the end-to-end joint learning of pose estimation and graph convolution to remove the dependence on external estimators. Second, we will design adaptive graph topology to dynamically model real occlusion distributions. Third, we will extend the gating mechanism to cross-modal and unsupervised person re-identification to broaden its practical application.

## Figures and Tables

**Table 1:** Comparative analysis on occluded datasets.

Method	Occluded-Duke		Occluded-REID	
	Rank-1	mAP	Rank-1	mAP
TransReID [11]	64.2	55.7	-	-
PCB [4]	42.6	33.7	41.3	38.9
PFD [29]	67.7	60.1	79.8	81.3
PVPM [24]	47.0	37.7	66.8	59.5
PAT [25]	64.5	53.6	81.6	72.1
HOReID [16]	55.1	43.8	80.3	70.2
MTIPE [30]	66.4	57.8	-	-
FED [31]	68.1	56.4	86.3	79.3
PGGA [26]	70.9	58.8	79.5	70.1
FRCE [28]	72.4	61.6	84.4	79.6
SCAT [32]	62.8	54.9	80.4	76.1
HEFormer [27]	73.2	61.4	-	-
SI-GCN (Ours)	69.8	59.2	86.9	79.8

**Table 2:** Comparative analysis with state-of-the-art methods on two partial datasets, namely Partial-REID [20] and Partial-iLIDS [21].

Method	Partial-REID		Partial-iLIDS	
	Rank-1	Rank-3	Rank-1	Rank-3
PAT [25]	88.0	92.3	76.5	88.2
FED [31]	83.1	-	-	-
VPM [33]	67.7	81.9	65.5	74.8
APN [34]	71.8	85.5	66.4	76.5
Swin [35]	82.3	89.6	63.9	75.6
PPCL [36]	83.7	88.7	71.4	85.7
HOReID [16]	85.3	91.0	72.6	86.4
KBFM [37]	68.7	82.2	64.1	73.9
TSA [38]	72.7	85.2	73.9	84.7
SI-GCN (Ours)	89.6	92.8	77.9	89.7

**Table 3:** Comparative analysis with state-of-the-art methods on two holistic datasets, Market-1501 [22] and DukeMTMC-reID [23].

Method	Market-1501		DukeMTMC	
	Rank-1	mAP	Rank-1	mAP
PCB [4]	92.3	77.4	81.8	66.1
VPM [33]	93.0	80.8	83.6	72.6
CDnet [39]	95.1	86.0	88.6	76.8
PH-GCN [40]	93.5	79.0	-	-
BOT [8]	94.1	85.7	86.4	76.4
SPReID [41]	92.5	81.3	-	-
MGCAM [42]	83.8	74.3	46.7	46.0
GPS [43]	95.2	87.8	88.2	78.7
PSE [44]	87.7	69.0	27.3	30.2
PGFA [20]	91.2	76.8	82.6	65.5
SI-GCN (Ours)	94.6	86.5	88.5	78.2

**Table 4:** Ablation study of core components on the Occluded-Duke and Occluded-REID datasets.

Index	STM	SIG	DCC	Occluded-Duke		Occluded-REID	
				Rank-1	mAP	Rank-1	mAP
1	×	×	×	42.6	33.7	41.3	38.9
2	√	×	×	48.5	38.2	52.3	45.6
3	√	√	×	67.2	56.5	82.6	73.9
4	√	√	√	69.8	59.2	86.9	79.8

## Data Availability

All data are included in the article. Please contact the corresponding author for further requests if necessary.
